# 5-alpha reductase inhibitors and MRI prostates: actively reducing prostate sizes and ambiguity

**DOI:** 10.1186/s12894-023-01235-4

**Published:** 2023-04-15

**Authors:** Ziting Wang, Kaiying Wang, Han Yang Ong, Woon Chau Tsang, Qing Hui Wu, Edmund Chiong

**Affiliations:** 1 Department of Urology, National University Hospital, Singapore, Singapore; 2 Department of Diagnostic Imaging, National University Hospital, Singapore, Singapore; 3 Department of Surgery, National University Singapore, Singapore, Singapore

**Keywords:** 5-alpha reductase inhibitor, Prostate cancer, MRI, Biopsy

## Abstract

**Background:**

Magnetic resonance imaging (MRI) scans are increasingly first-line investigations for suspected prostate cancer, and essential in the decision for biopsy. 5-alpha reductase inhibitor (5-ARI) use has been shown to reduce prostate size and prostate cancer risk. However, insufficient data exists on how 5-ARI use affects MRI findings and yield of biopsy. This study explores the differences in imaging and prostate cancer diagnoses between patients receiving and not receiving 5-ARI therapy.

**Methods:**

From 2015 to 2020, we collected retrospective data of consecutive patients undergoing prostate biopsy at one centre. We included patients who were biopsy-naïve, had prior negative biopsies, or on active surveillance for low-grade prostate cancer. Clinical and pathological data was collected, including 5-ARI use, Prostate Imaging Reporting and Data System (PIRADS) classification and biopsy results.

**Results:**

351 men underwent saturation biopsy with or without targeted biopsies. 54 (15.3%) had a history of 5-ARI use. On mpMRI, there was no significant difference between the 5ARI and non-5-ARI groups in PIRADS distribution, number of lesions, and lesion location. Significantly fewer cancers were detected in the 5-ARI group (46.3% vs. 68.0%; *p* < 0.01). There were no significant differences in PIRADS distribution in 5-ARI patients with positive and negative biopsy.

**Conclusion:**

Our study found significant differences in biochemical, imaging and biopsy characteristics between 5-ARI and non-5-ARI groups. While both groups had similar PIRADS distribution, 5-ARI patients had a lower rate of positive biopsies across all PIRADS categories, which may suggest that the use of 5ARI may confound MRI findings. Further studies on how 5-ARI therapy affects the imaging characteristics of prostate cancer should be performed.

**Supplementary Information:**

The online version contains supplementary material available at 10.1186/s12894-023-01235-4.

## Background

There has been increasing recognition of the utility of multiparametric magnetic resonance imaging (mpMRI) in the diagnosis of prostate cancer. With the emergence of several large studies showing MRI before biopsy was superior to biopsy alone [1, 2], both the European Association of Urology (EAU) and American Urological Association (AUA) guidelines were updated to recommend mpMRI prior to biopsy, both for men without previous history of biopsy and with a previous negative biopsy but persistent clinical suspicion of prostate cancer [3, 4].

5-alpha reductase inhibitors (5ARIs) are one of the first-line drugs used to treat benign prostate hyperplasia (BPH). BPH is a common affliction, with 50% of men developing pathological BPH at 51–60 years of age [5]. The effects of 5ARIs on the prostate have been extensively studied—they are known to reduce prostate volume and prostate specific antigen (PSA) levels [6], and reduce the risk of prostate cancer [7, 8]. They may also be associated with an increased risk of high-grade prostate cancer [9], although other studies have contested this [10].

The effect of 5ARIs on mpMRI, however, is not yet well-established. Giganti et al. [11] found that dutasteride decreased visibility of prostate cancer on diffusion-weighted imaging (DWI) in mpMRI, but had no effect on T2-weighted imaging [12]; Starobinets et al. [13] however found that 5ARIs reduced cancer discrimination on T2-weighted imaging, but improved discrimination with functional MR measures. Given the increasing uptake of mpMRI as a screening and diagnostic tool for prostate cancer, and the high prevalence of BPH and 5ARI use, the effects of 5ARIs on mpMRI is clearly an important question to answer.

The aim of this study was thus to explore the differences in mpMRI imaging and prostate cancer diagnoses in clinical practice, between patients receiving and not receiving 5ARI therapy.

## Methods

In this single-centre retrospective observational study conducted from September 2015 to October 2020, patients underwent a saturation biopsy via trans-perineal route. If a Prostate Imaging Reporting and Data System version 2 (PIRADS) 3 and above lesion was seen on MRI, targeted biopsies via MRI-Transrectal ultrasound (TRUS) fusion prostate biopsy were also obtained. Information on clinical history, use of 5ARI, duration of 5ARI use, lesion characteristics, PIRADS classification and follow up was collected.

### Patients, imaging, and biopsy data

From September 2015 to October 2020, patients who underwent trans-perineal prostate biopsy from a single-centre institution at the National University Hospital, Singapore were recruited into a retrospective observational study. Waiver of consent was obtained, as approved by the Domain Specific Review Board of the National Healthcare group, Singapore. Information on clinical data (lower urinary tract symptoms, digital rectal examination (DRE), family history), lesion characteristics on mpMRI, PIRADS classification and follow-up was collected.

The inclusion criteria consisted of all men presenting with lower urinary tract symptoms with PSA > 4, or PIRADS 3 and above, regardless of whether they had previous prostate biopsy.

All the patients underwent mpMRI before biopsy. Image acquisition and processing were performed according to standard clinical mpMRI protocols in the Department of Radiology, National University Hospital, Singapore. T2W and DWI sequences were evaluated based on the PIRADS version 2 developed by the European Society of Urogenital Radiology [14]. There were 2 MRI readers, each with more than 10 years of experience in uro-radiology. The MRI protocol can be found in Additional file 1.

If a PIRADS 3 and above lesion was seen on MRI, targeted prostate biopsies via MRI-TRUS fusion prostate biopsy were also obtained. The index lesion was defined as the lesion with the highest PIRADS score. If there was more than 1 lesion with the highest PIRADS score, the larger one was selected as the index lesion. Otherwise, men with PIRADS 1 or 2 lesions had only saturation biopsies performed. Saturation biopsies were performed with a standardised saturation biopsy template as a guide. Procedures were done by one of two uro-oncologists with more than 10 years of experience in performing prostate biopsies. The exact biopsy protocol can be found in Additional file 1.

Biopsy samples were processed through a single pathology laboratory, and reviewed by a single uro-pathologist with more than 10 years of experience in uro-pathology. Any discrepancies in histology were re-reviewed in multidisciplinary uropathology meetings before a final grade and diagnosis were assigned.

### Serum biomarkers

PSA and prostate health index (PHI) were taken prior to biopsies, and at least four weeks after any prostate manipulation, urinary tract infection, and/or catheter insertion/removal. PSA density (PSAD) was derived by dividing PSA by the prostate volume, which was measured in mpMRI. In the 5ARI group, PSA values were multiplied by a correction factor of 2 to obtain corrected PSA [15].

### Statistical analysis

Comparative analyses of the 5ARI and non-5ARI groups were conducted using the Chi-squared test for categorical variables, and the independent 2-tailed t-test for continuous variables. Statistical analyses were performed using Stata STATA IC13.1 (StataCorp, 4905 Lakeway, College Station, TX, 77,845, USA). Statistical significance was considered when the two-sided *p* value was less than 0.05.

Subgroup analyses was performed within the 5ARI group, according to the duration of 5ARI use and eventual diagnosis.

## Results

351 patients were included, of which 54 (15.4%) had a history of 5ARI use, and 297 (84.6%) were 5ARI-naïve. Clinical demographics and serum biomarkers of the two groups are summarised in Table 1. The mean age of the 5ARI group was marginally higher than the non-5ARI group (67.6 vs. 65.6; *p* = 0.0460).

**Table 1:** Clinical demographics and serum biomarkers

	5ARI users	Non-5ARI users
Number of patients	54	297
Age	67.6 ± 6.21	65.6 ± 6.85
Prostate volume	64.9 ± 33.5	48.1 ± 21.8
Indication for biopsy		
No prior biopsies	30 (55.6%)	159 (53.5%)
Active Surveillance	3 (5.56%)	61 (20.5%)
Prior negative biopsy	21 (38.9%)	77 (25.9%)
Abnormal digital rectal examination	13 (24.1%)	72 (24.2%)
PSA	11.0 ± 8.33 (22.0 ± 16.7 corrected)	9.41 ± 8.41
PSA density	0.194 ± 0.142 (0.387 ± 0.284 corrected)	0.214 ± 0.177
PHI	35.2 ± 16.2	47.8 ± 27.1
PHI density	0.667 ± 0.460	1.18 ± 0.849
Free: Total PSA	0.127 ± 0.074	0.165 ± 0.079

Prostate-specific characteristics differed between the two groups. Prostate volume (64.9 ± 6.21 vs. 48.1 ± 6.85, *p* < 0.01), corrected PSA (22.0 ± 16.7 vs. 9.41 ± 8.41; *p* < 0.01), and corrected PSA density (0.387 ± 0.284 vs. 0.214 ± 0.165; *p* < 0.01) were significantly higher in the 5ARI group. PHI (35.2 ± 16.2 vs. 47.8 ± 27.1, *p* < 0.01) and PHI density (0.667 ± 0.460 vs. 1.18 ± 0.847, *p* < 0.01), however, were significantly lower in the 5ARI group.

Regarding imaging characteristics on MRI (Table 2), there was no significant difference in distribution of index lesions’ PIRADS score between the two groups, χ^2^(3, N = 351) = 4.06, *p* = 0.255. There were also no significant differences in the number of lesions between 5ARI users and non-5ARI users, χ^2^(5, N = 351) = 5.60, *p* = 0.347. The location of the index lesion was also comparable between the two groups, χ^2^(2, N = 328) = 0.203, *p* = 0.904.

**Table 2:** Imaging characteristics

	5ARI users	Non-5ARI users
Prostate volume	64.9	48.1
*Index PIRADS lesion*		
1–2	3 (5.56%)	27 (9.09%)
3	23 (42.6%)	88 (29.6%)
4	21 (38.9%)	145 (48.8%)
5	7 (13.0%)	37 (12.5%)
*Number of lesions*		
0	3 (5.55%)	18 (6.06%)
1	34 (63.0%)	186 (62.6%)
2	12 (22.2%)	69 (23.2%)
3	3 (5.55%)	19 (6.39%)
4	1 (1.85%)	5 (1.68%)
5	1 (1.85%)	0
*Location of index lesion*		
Peripheral zone	35 (68.6%)	192 (69.3%)
Transitional zone	16 (31.4%)	84 (30.3%)
Anterior	0 (0%)	1 (0.4%)

The histopathological findings post-biopsy are detailed in Table 3. Overall, rate of detection of prostate cancer was significantly lower in the 5ARI group (46.3%) compared to the non-5ARI group (68.0%), χ^2^(1, N = 351) = 0.203, *p* < 0.01. There was also a lower absolute rate of detection of clinically significant prostate cancer in the 5ARI group (31.5% vs. 45.5%), although this difference was statistically insignificant, χ^2^(1, N = 351) = 3.63, *p* = 0.0566. The proportion of clinically significant cancers out of all cancers detected was comparable between the two groups (68.0% vs. 66.8%), χ^2^(1, N = 227) = 0.0137, *p* = 0.907.

**Table 3:** Histopathological findings

	5ARI users				Non-5ARI users			
	PIRADS 1–2 or no lesions (n = 3)	PIRADS 3 (n = 23)	PIRADS 4 (n = 21)	PIRADS 5 (n = 7)	PIRADS 1–2 or no lesions (n = 27)	PIRADS 3 (n = 88)	PIRADS 4 (n = 145)	PIRADS 5 (n = 37)
Any prostate cancer detected	0	8 (34.8%)	12 (57.1%)	5 (71.5%)	10 (37.0%)	38 (43.1%)	118 (81.4%)	36 (97.3%)
Prostate cancer detected on targeted biopsies	0	5 (21.7%)	10 (47.6%)	4 (57.1%)	0	26 (29.5%)	93 (64.1%)	36 (97.3%)
Prostate cancer detected on saturation biopsies	0	7 (30.4%)	10 (47.6%)	4 (57.1%)	10 (37.0%)	32 (36.4%)	112 (77.2%)	31 (83.8%)
Clinically significant prostate cancer detected	0	2 (8.70%)	10 (47.6%)	5 (71.5%)	4 (14.8%)	18 (20.4%)	82 (56.6%)	31 (83.8%)

Subgroup analysis was performed on the 5ARI group by duration of 5ARI use, specifically less than 6 months, 6 months to 1 year, and more than 1 year (Table 4). 51/54 patients (94.4%) were included, as duration of 5ARI use was not available for 3 patients.

**Table 4:** 5ARI users stratified by duration of 5ARI use

Duration of 5ARI use	MRI findings				Prostate cancer detected	No prostate cancer detected	Clinically significant cancer detected	No clinically significant cancer
	PIRADS 0–2	PIRADS 3	PIRADS 4	PIRADS 5				
Less than 6 months (n = 16)	0	8 (50%)	7 (43.8%)	1 (6.2%)	5 (31.3%)	11 (68.7%)	2 (12.5%)	14 (87.5%)
6 months to 1 year (n = 8)	1 (12.5%)	4 (50%)	3 (37.5%)	0	2 (25%)	6 (75%)	0	8 (100%)
More than 1 year (n = 27)	2 (7.41%)	10 (37.0%)	9 (33.3%)	6 (22.2%)	17 (63.0%)	10 (27.0%)	14 (51.9%)	13 (48.1%)

Between the three groups, there was no significant difference in distribution of index lesions PIRADS score, χ^2^(6, N = 51) = 5.60, *p* = 0.468. The subgroup of patients with 5ARI use of longer than 1 year had a higher rate of detection of prostate cancer (63.0%), although this was statistically insignificant, χ^2^(2, N = 51) = 5.91, *p* = 0.0521. There was no difference in rate of clinically significant cancers, χ^2^(2, N = 51) = 3.63, *p* = 0.0566.

To identify predictive factors for prostate cancer in patients taking 5ARIs, subgroup analysis was also performed for the 5ARI group based on eventual diagnosis post-biopsy (Table 5). There were no significant differences found in corrected PSA (19.6 ± 8.46 vs. 24.1 ± 21.3, *p* = 0.303) and corrected PSAD (0.456 ± 0.282 vs. 0.329 ± 0.278, *p* + 0.102) between the cancer and cancer-free groups. However, the group diagnosed with cancer had significantly higher PHI scores (43.5 ± 18.7 vs. 30.0 ± 12.2, *p* = 0.0403) and PHID (1.03 ± 0.530 vs. 0.437 ± 0.193, *p* < 0.01).

**Table 5:** 5ARI users stratified by eventual diagnosis

	Overall cancers (n = 25)	No cancers (n = 29)	Clinically significant cancers (n = 17)	No Clinically significant cancers (n = 37)
Patients with prior negative biopsy	7 (28.0%)	14 (48.3%)	7 (41.2%)	14 (37.8%)
PSA	9.80	12.05	10.45	11.26
Corrected PSA	19.6	24.1	20.9	22.52
PSA density	0.228	0.164	0.254	0.166
Corrected PSA density	0.456	0.329	0.507	0.332
Abnormal DRE	6 (24.0%)	7 (24.1%)	4 (23.5%)	9 (24.3%)
PHI	43.5	30.0	53.2	31.8
PHI density	1.03	0.437	1.34	0.538

Similarly, between the group diagnosed with clinically significant cancer and the group with either clinically insignificant cancer or no cancer, there were no significant differences in corrected PSA (20.9 ± 9.33 vs. 22.5 ± 19.2, *p* = 0.676) and corrected PSAD (0.507 ± 0.319 vs. 0.332 + 0.253, *p* = 0.0576). However, the group with clinically significant cancer had significantly higher PHI scores (53.2 ± 17.6 vs. 31.8 + 13.7, *p* = 0.0499) and PHID (1.34 ± 0.468 vs. 0.538 ± 0.334, *p* = 0.0147) than the group with no clinically significant cancer.

A further subgroup analysis was performed on the 5ARI group with index lesion of PIRADS 3 (Table 6). Between the group found to have cancer and the cancer-free group, there were no significant differences in corrected PSA (17.2 ± 5.90 vs. 30.0 ± 26.7, *p* = 0.0935), corrected PSAD (0.347 ± 0.168 vs. 0.400 ± 0.366, *p* = 0.645), PHI (41.8 ± 17.8 vs. 35.0 ± 11.8, *p* = 0.473), and PHID (0.923 ± 0.532 vs. 0.487 ± 0.218, *p* = 0.140). Between the groups with and without clinically significant cancer, the corrected PSA (17.3 ± 6.90 vs. 26.3 ± 23.3, *p* = 0.268) and corrected PSAD (0.364 ± 0.316 vs. 0.383 ± 0.315, *p* = 0.950) were comparable as well.

**Table 6:** 5ARI users with PIRADS 3 index lesion

	Overall cancers (n = 8)	No cancers (n = 15)	Clinically significant cancers (n = 2)	No Clinically significant cancers (n = 21)
Patients with prior negative biopsy	1 (12.5%)	8 (53.3%)	1 (50.0%)	8 (38.1%)
PSA	8.59	15.01	8.64	13.17
Corrected PSA	17.2	30.0	17.3	26.3
PSA density	0.174	0.200	0.182	0.191
Corrected PSA density	0.347	0.400	0.364	0.383
Abnormal DRE	0 (0%)	4 (26.7%)	0 (0%)	4 (19.0%)
PHI	41.8	35.0	–	37.4
PHI density	0.923	0.487	–	0.643

## Discussion

Current evidence reflects that 5ARIs have essential roles in the prostate cancer prevention. The prostate cancer prevention trial (PCPT) [9] and Reduction by Dutasteride of Prostate Cancer Events (REDUCE) trial [16] both demonstrated significant reduction in prostate cancer incidences. Although there was an initial concern of a greater prevalence of high Gleason grade cancers, this controversy was disputed in the long term follow up of PCPT [17], which showed no long term risk of increased death from prostate cancers.

Considering the growing pool of evidence showing that prostate cancer has similar incidence in 5ARI users and 5ARI-naïve patients, the question remains as to whether the ability to detect cancer is equivalent in those two groups. The PROMIS [2], PRECISION [1], 4M [18] and MRI-FIRST [19] trials, amongst others, have proven the role of MRI scans to detect more clinically significant cancers and less clinically insignificant cancer whilst enabling a percentage of men to avoid biopsies. Hence, international guidelines have moved to adopt MRI scans as an early evaluation for men with suspicions of prostate cancer.

Kim et al. [20] first evaluated the effect of 5ARI treatment on prostate cancer and clinically significant prostate cancer detection in patients undergoing MRI-TRUS fusion biopsy. They concluded that 5-ARI use had no significant association with prostate cancer detection, and exposure to 5-ARI may not impair mpMRI performance. However, it should be noted that the mean number of cores taken was 14, and in the setting of sizable prostates that ranged from 28 to 57 c in volume, there may have been some degree of under-sampling and false negative results.

Medina et al. [21] highlighted a concern of 5ARI use being associated with MRI invisible prostate cancer. However, the study did not make a distinction between clinically significant and insignificant prostate cancer, and 65% of the prostate cancers detected yielded Gleason 3 + 3 histology. Prostate volume may be a confounder that could result in this association. Within our series, we reviewed the 5ARI users with an MRI reading of PIRADS 2 or less and did not find any prostate cancer diagnosed on biopsies. It is equally pertinent to evaluate if the use of 5ARI had any impact on the interpretation of malignancy on MRI.

In our dataset, while the 5ARI and non-5ARI groups are largely comparable, there is a lower proportion of 5ARI users on active surveillance (5.56%) compared to the non-5ARI users (20.5%). The 5ARI users also had a larger prostate size (64.9 ± 33.5 ml) than the non-5ARI users (48.1 ± 21.8 ml). It is true that there are intrinsic differences in the 2 groups of 5ARI users versus non-5ARI users, with proportionately fewer 5ARI patients being on active surveillance as compared to the non-5ARI users. However, as this is an observational study, we are unable to draw any definitive conclusion about the cause and effect. Similarly, regarding the difference in size of the prostate, we believe this is in part necessarily due to a baseline difference rather than cause and effect. The 5ARI group would have been on 5ARIs due to large prostate causing lower urinary tract symptoms, while those not on 5ARIs likely had no size indication for 5ARI.

It should be noted that the evidence recommending active surveillance for low-grade prostate cancer has been growing in the last decade. Starobinets et al. [13] suggested that there was an increase in homogeneity of benign and malignant peripheral zone prostatic tissues with 5-ARI exposure, hence facilitating better discrimination of low-grade prostate cancer from benign tissues with multiparametric MRI. However, it should be noted that Starobinets’ cohort involved Gleason 3 + 3 patients, many of whom may have been managed with active surveillance instead of radical treatment in the contemporary world. An important research question to evaluate in larger prospective studies would be the effect of 5ARI on the MRI appearance of intermediate or high-risk cancers.

We also explored the additional effects that 5ARI use may have on the detection of cancer in differing locations. A presiding hypothesis was that 5ARI use may result in a greater reduction of transitional volume compared to peripheral zone volume as most of the prostate tissue enlargement in benign prostatic hyperplasia occurs in the transitional zone [22]. Hence, 5ARI use may enable improved detection rate for tumours in the transitional zone.

A review of our patient cohort with PIRADS 3 and above lesions did not find any statistical differences in the prevalence of lesions detected in the transitional zone or peripheral zones between 5ARI users and non-users. However, 5ARI use was associated with a lower detection rate of overall and clinically significant cancers. This was in contrast in Giganti et al. [11] who evaluated Apparent Diffusion Coefficient (ADC) changes in lesions of men who received 6 months of 5ARIs. They concluded that 5ARI use was associated with increased tumour ADC and suggested that physicians should consider a lower biopsy threshold for men on 5ARIs. ADC plays a much larger role in the evaluation of lesions in the peripheral zone compared to the transitional zone. Furthermore, other indices such as DWI and dynamic contrast enhancement parameters also contribute to a global assessment of the lesion, hence it may be more prudent to evaluate the effect of 5ARIs on the PIRADS reading rather than individual parameters.

To our knowledge this study is the first to identify factors that could suggest the presence of clinically significant prostate cancer amongst 5ARI users. It also sheds light on the interpretation of PIRADS lesions within this population. We recognize that the sample size and retrospective nature are considerable limitations. However, it should be noted that the concurrent use of multiparametric MRI scans of the prostate and a saturation biopsy template provides a comprehensive assessment of the prostate, and high detection rates of any cancer present.

## Conclusions

In conclusion, there were significant differences in imaging characteristics between 5-ARI and non-5ARI users. Patients receiving 5-ARI therapy had significantly higher prostate volume, corrected PSA and corrected PSA density, as well as significantly lower PHI density. Notably, 5-ARI patients had a lower rate of positive biopsies across all PIRADS categories, which may suggest that the use of 5ARI may confound MRI findings. Higher PHI levels may be predictive of clinically significant cancer in 5-ARI users. Further studies on how 5-ARI therapy affects imaging characteristics of prostate cancer should be performed.

## Supplementary Information

**Additional file 1**: MRI and biopsy protocol.

## Data Availability

The datasets used and/or analysed during the current study are available from the corresponding author on reasonable request.

## Abbreviations

5ARI: 5-Alpha reductase inhibitor
ADC: Apparent diffusion coefficient
AUA: American Urological Association
BPH: Benign prostate hyperplasia
DRE: Digital rectal examination
EAU: European Association of Urology
MRI: Magnetic resonance imaging
mpMRI: Multiparametric magnetic resonance imaging
PHI: Prostate health index
PIRADS: Prostate imaging reporting and data system
PSA: Prostate specific antigen
PSAD: Prostate specific antigen density
TRUS: Transrectal ultrasound
