# Supplementary material for: 5-alpha reductase inhibitors and MRI prostates: actively reducing prostate sizes and ambiguity
Source: BMC Urol. 2023 Apr 15;23:61. doi: 10.1186/s12894-023-01235-4 (PMC10105450; doi:10.1186/s12894-023-01235-4)
Supplement: Supplementary file 1 — Additional file 1: MRI and biopsy protocol. [file 12894_2023_1235_MOESM1_ESM.docx]

MRI and prostate biopsy protocols

All men underwent a 3-Tesla multi-parametric MRI performed to standards established by the European Society of Uroradiology. No endorectal coils were used. Images were reviewed by 2 board-certified uroradiologist with more than 10 years experienced (HT) and given a score using the PIRADS V2 scoring system 1–5. mpMRI images were uploaded onto the transperineal prostate biopsy platform, iSR’obot Mona Lisa^TM^ (Biobot Surgical, Singapore). A biplane transducer (BK 8848, 9 MHz, BK Medical, Analogic Ultrasound Group) was used to perform intra-operative ultrasound imaging of the prostate, and a software fusion programme (UroFusion^TM^, Biobot Surgical, Singapore). was used to fuse the pre-biopsy MRI mapping to the intraoperative ultrasound images. The operator for the biopsies were 2 uro-oncologists with more than 10 years experienced at performing prostate biopsies. Saturation biopsies were done in the operating theatre, under general anaesthesia, with an induction dose of Ceftriaxone or Gentamycin for antibiotics prophylaxis. A saturation biopsy template was used to guide biopsy. The number of cores ranged from 10 to 65, with a median number 25.
